# MicroRNA‐9 inhibits growth and invasion of head and neck cancer cells and is a predictive biomarker of response to plerixafor, an inhibitor of its target CXCR4

**DOI:** 10.1002/1878-0261.12352

**Published:** 2018-10-25

**Authors:** Hersi Mohamed Hersi, Nina Raulf, Joop Gaken, Najeem'deen Folarin, Mahvash Tavassoli

**Affiliations:** ^1^ Department of Molecular Oncology King's College London UK; ^2^ Department of Haematological Medicine The Rayne Institute King's College London UK; ^3^ King's College London Hospital NHS Foundation Trust UK

**Keywords:** CXCL12, CXCR4, head and neck cancer, MiR‐9, plerixafor, tumour invasion

## Abstract

Head and neck squamous cell carcinomas (HNSCC) are associated with poor morbidity and mortality. Current treatment strategies are highly toxic and do not benefit over 50% of patients. There is therefore a crucial need for predictive and/or prognostic biomarkers to allow treatment stratification for individual patients. One class of biomarkers that has recently gained importance are microRNA (miRNA). MiRNA are small, noncoding molecules which regulate gene expression post‐transcriptionally. We performed miRNA expression profiling of a cohort of head and neck tumours with known clinical outcomes. The results showed miR‐9 to be significantly downregulated in patients with poor treatment outcome, indicating its role as a potential biomarker in HNSCC. Overexpression of miR‐9 in HNSCC cell lines significantly decreased cellular proliferation and inhibited colony formation in soft agar. Conversely, miR‐9 knockdown significantly increased both these features. Importantly, endogenous CXCR4 expression levels, a known target of miR‐9, inversely correlated with miR‐9 expression in a panel of HNSCC cell lines tested. Induced overexpression of CXCR4 in low expressing cells increased proliferation, colony formation and cell cycle progression. Moreover, CXCR4‐specific ligand, CXCL12, enhanced cellular proliferation, migration, colony formation and invasion in CXCR4‐overexpressing and similarly in miR‐9 knockdown cells. CXCR4‐specific inhibitor plerixafor abrogated the oncogenic phenotype of CXCR4 overexpression as well as miR‐9 knockdown. Our data demonstrate a clear role for miR‐9 as a tumour suppressor microRNA in HNSCC, and its role seems to be mediated through CXCR4 suppression. MiR‐9 knockdown, similar to CXCR4 overexpression, significantly promoted aggressive HNSCC tumour cell characteristics. Our results suggest CXCR4‐specific inhibitor plerixafor as a potential therapeutic agent, and miR‐9 as a possible predictive biomarker of treatment response in HNSCC.

**Abbreviations**
BD: Becton Dickinson Bioscience
G‐CSF: granulocyte colony‐stimulating factor
HNSCC: head and neck squamous cell carcinoma
HSCs: haematopoietic stem cells
MTT: 3‐(4,5‐dimethylthiazol‐2‐yl)‐2,5‐diphenyltetrazolium bromide
Polyhema: poly(2‐hydroxyethylmethacrylate)
SDF‐1: stromal cell‐derived factor 1

## Introduction

Head and neck squamous cell carcinoma (HNSCC) is the sixth most common cancer in the world with approximately 650 000 new cases diagnosed each year (Ferlay *et al*., 2010; Li *et al*., 2011; Raulf *et al*., 2014). HNSCC are a group of heterogeneous tumours of the oral cavity, oropharynx, larynx or hypopharynx that usually present with locally advanced disease (Suh *et al*., 2014). Several risk factors have been correlated with HNSCC including tobacco, alcohol, genetic susceptibility and viral infection (Ragin *et al*., 2007). Typical treatment consists of surgery and radiotherapy; however, patients generally develop resistance, as a result, 5‐year survival rates of HNSCC patients are around 40–50% (Mehanna and Ang, 2012). Therefore, predictive biomarkers and targeted therapies are required to enable individualisation of treatment and improve outcomes.

MicroRNA (miRNA) are small (20–22nt), tissue‐specific, noncoding RNA molecules that have an important post‐transcriptional regulatory role in gene expression. Genomewide miRNA profiling studies performed on various cancer types such as breast (Iorio *et al*., 2005), glioblastoma (Chan *et al*., 2005), hepatocellular carcinoma (Murakami *et al*., 2006) and lung (Yanaihara *et al*., 2006) amongst others (reviewed Calin and Croce, 2006) showed that miRNA profiles in cancers can be used to differentiate between disease subtypes and predict patient survival and treatment response (Lu *et al*., 2005). MiRNA expression in cancer was globally downregulated compared to normal tissues and could be used to discriminate between different developmental lineage and differentiation status. Expression profiling in prostate cancer has shown that a panel of nine miRNA could discriminate between neoplastic and normal tissue (Carlsson *et al*., 2011). In cervical cancer‐specific miRNA expression profiles were predictive of stage (downregulation of seven miRNA), metastasis (six miRNA) and prognosis (two miRNA) (Huang *et al*., 2012). Moreover, miRNA expression profiling performed in lung cancer showed that specific miRNA signatures were able to discriminate between lung cancer and normal lung tissues and were also able to differentiate between tumours with different prognosis (Yanaihara *et al*., 2006). MiRNA have potential diagnostic and prognostic roles as biomarkers in a variety of cancers including HNSCC (Hui *et al*., 2010, 2016; Summerer *et al*., 2015).

MiR‐9 has emerged in recent years as an important miRNA in various cancer types (Cekaite *et al*., 2012; Liu *et al*., 2012; Song *et al*., 2014; Sun *et al*., 2009; Xu *et al*., 2014). MiR‐9 is encoded on chromosomes 1 (miRNA‐9‐1), 5 (miRNA‐9‐2) and 15 (miRNA‐9‐3) all of which give rise to the same mature miRNA. All three genomic loci have CpG islands, and hypermethylation of miR‐9 loci occurs in several different cancer types including breast, lung, colon, melanoma, acute lymphoblastic leukaemia and HNSCC (Bandres *et al*., 2009; Hsu *et al*., 2009; Lujambio *et al*., 2008; Roman‐Gomez *et al*., 2009). MiR‐9 expression and its role seem to vary in different tumour types (Fenger *et al*., 2014; Hildebrandt *et al*., 2010; Lu *et al*., 2012, 2014a,2014b; Sun *et al*., 2013; Yu *et al*., 2014). Several target genes of miR‐9 have been identified including E‐cadherin (Ma *et al*., 2010), NFκB1 (Bazzoni *et al*., 2009) and CXCR4 (He *et al*., 2017).

CXCR4 is a 352 amino acid rhodopsin‐like alpha G‐protein‐coupled receptor that exclusively binds the CXCR4 chemokine stromal cell‐derived factor 1 (SDF‐1) also known as CXCL12 (Busillo and Benovic, 2007). Knockout studies of either CXCL12 or CXCR4 in mice have shown similar phenotypic consequences, late gestational lethality, bone marrow colonisation, cardiac septum formation and defects in B‐cell lymphopoiesis indicating that CXCR4 is essential for development (Nagasawa *et al*., 1996; Zou *et al*., 1998). Moreover, CXCR4 is important in classical chemokine receptor response in adults as well as neutrophil maturation (Machado *et al*., 2016). CXCR4 has been implicated in tumour metastasis in several tumour models including melanoma (Neagu *et al*., 2015), prostate (Lee *et al*., 2014) and neuroblastoma metastasis (Mühlethaler‐Mottet *et al*., 2015) and has been suggested as a biomarker for HNSCC with high metastatic potential (Albert *et al*., 2013). Plerixafor is the only approved drug that targets CXCR4 and CXCR7. It is used to mobilise haematopoietic stem cells (HSCs) from the bone marrow into the peripheral blood circulation in the 30–40% of lymphoma and multiple myeloma patients who do not respond to the effects of granulocyte colony‐stimulating factor (G‐CSF) alone (Wagstaff, 2009). Currently, using plerixafor in combination with conventional therapy is in clinical trials for other cancers such as prostate and cervical cancer (Chaudary *et al*., 2017; Conley‐LaComb *et al*., 2016) but not HNSCC. Additionally, plerixafor is the only approved drug for selecting haematological stem cells for autologous stem cell transplantation (Anonymous, 2007).

We performed miRNA expression profiling of a panel of HNSCC tumours and found that miR‐9 was significantly downregulated in a group of HNSCC patients with known negative clinical outcome after conventional treatment. We subsequently performed functional studies by modulating miR‐9 expression in a panel of HNSCC cell lines. Knockdown of miR‐9 in HNSCC cells resulted in increased proliferation, cell cycle progression, increased invasion and enabled colony formation in soft agar. By luciferase reporter assay, we confirmed CXCR4 to be a direct target of miR‐9 in HNSCC. The knockdown of miR‐9 or overexpression of CXCR4 in HNSCC cell lines had similar consequences on tumour cell behaviour. Importantly, the effect of miR‐9 knockdown could be abrogated by treatment of cells with CXCR4‐specific inhibitor plerixafor.

This study demonstrates a clear tumour suppressor role for miR‐9 in HNSCC and suggests that the potential oncogenic effects of miR‐9 knockdown in HNSCC are mediated through targeting CXCR4. Furthermore, we demonstrate that miR‐9 may be a potential biomarker for response of HNSCC to the CXCR4 inhibitor plerixafor.

## Materials and methods

### Cell lines and culture

The cell lines HSC3 and HSC3M3 were gifts from Kazuya Tominaga, Department of Oral Pathology, Osaka Dental University (Hirakata, Osaka, Japan). The H357 cell line was a gift from Stephen Prime, Department of Oral and Dental Science, University of Bristol (Bristol, UK). HN5 was obtained from Professor Barry Gusterson, Department of Pathology, University of Glasgow, UK, and HN30 from Andrew Yeudall, Philips Institute of Oral and Craniofacial Molecular Biology, Virginia Commonwealth University, Richmond, Virginia, USA. HEK293T cells were provided by Lucas Chan, Rayne Institute, King's College London, UK. MDA‐MB‐231 was obtained from Joy Burchell, Breast Cancer Biology Group, King's College London, UK, to act as a positive control for the invasion assay. Five head and neck cancer cell lines were profiled for expression of miR‐9. Two cell lines with high (HN30 and H357) and two cell lines with low (HSC3 and HN5) miR‐9 expression were selected for further investigation. Cell lines were authenticated by STR profiling. STR profiles were obtained using the Promega Powerplex 16 assay according to the manufacturer's procedure. STR profiles were compared to published profiles (web.expasy.org/cellosaurus/). For H357, there is no published STR profile but a search of the ATCC database did not find significant homology with other cell lines.

All cell lines except H357 were cultured in DMEM supplemented with 10% FBS, 50 μg·mL^−1^ streptomycin, 100 μg·mL^−1^ penicillin and 1 mm sodium pyruvate. H357 cells were cultured in DMEM‐F12 supplemented with 10% FBS, 4 mm l‐glutamine, 69 nm hydrocortisone, 5 μg·mL^−1^ streptomycin, 5 μg·mL^−1^ penicillin and 1 mm sodium pyruvate. The CXCR4‐specific ligand CXCL12 (Bio‐Techne) was supplemented to appropriate cell media at a final concentration of 100 ng·mL^−1^ whereas the CXCR4‐specific inhibitor, plerixafor (Cambridge Bioscience, Cambridge, UK), was supplemented to appropriate cell media at a final concentration of 500 ng·mL^−1^.

### Plasmids and transfection

MiR‐9 was PCR‐amplified from HeLa genomic DNA as a 977‐bp fragment using the following primers: GATGCGCCCTCGATCTTC and CTGTGGGAAAGTGTTCAC. The PCR products were TA cloned into pCR2.1 (Invitrogen, Carlsbad, CA, USA), and the sequence was verified with M13 forward and reverse primers and two internal primers F:CAAGTTGACCAGTGCCGTTC and R:CTCGGTACCCCACGAAGTG. A scrambled sequence was used as a control for the miR‐9 constructs. Calcium phosphate precipitation was used to transfect the HEK293T to generate miR‐9 knockdown and overexpression retrovirus as previously described (Suh *et al*., 2015). CXCR4 overexpression/knockdown constructs were gifts from Gilbert Fruhwirth, Faculty of Life Sciences and Medicine King's College London. HA‐CXCR4 overexpression retrovirus and shRNA CXCR4 knockdown lentivirus were generated in HEK293T after calcium phosphate transfection as previously described (Suh *et al*., 2015). At 24, 36 and 48 h after transfection, viral supernatants were harvested and filtered through a 0.45‐μm filter and supplemented with 5 μg·mL^−1^ polybrene. Cells were infected with the virus overnight, and the infected cells were selected based on their newly acquired antibiotic resistance.

### Luciferase gene reporter assay

5 × 10^3^ cells were seeded per well on a 96‐well plate overnight, and the cells were infected with MISSION^®^ CXCR4 3′UTR Lenti GoClone (Sigma, St. Louis, MO, USA) for 48 h. The activity of the luciferase was determined using the Dual‐Glo Luciferase Assay System (Promega, Madison, WI, USA) according to the manufacturer's instructions. Firefly bioluminescence was used as experimental read‐out with Renilla bioluminescence as an internal control. Samples were measured on a Veritas Luminometer (Turner Biosystems, Sunnyvale, CA, USA). Untransfected cells served as negative control.

### RNA extraction and qRT–PCR

Total RNA was isolated from 2 × 10^6^ cells using Trizol (Invitrogen). cDNA was generated from 10 ng of RNA with miR‐9 and RNU6B primers (Applied Biosystems, Foster City, CA, USA) and the TaqMan miRNA Reverse Transcription Kit (Applied Biosystems). Whereas for CXCR4 expression, cDNA was synthesised from 1.5 μg RNA with CXCR4 (Integrated DNA Technologies, Skokie, IL, USA) and YWHAZ primers (Sigma) and the SuperScript III Reverse Transcriptase kit (ThermoFisher). Quantitative PCR was performed on the Corbett Rotor‐Gene 6000 with TaqMan Universal Mastermix‐No UNG (Applied Biosystem) for miR‐9 and 5x EvaGreen qPCR mix for CXCR4 (Solis Biodyne, Tallinn, Estonia). Delta‐Ct was calculated after normalising to RNU6B for miR‐9 and YWHAZ for CXCR4. miR‐9 primers were purchased from Applied Biosystems, and CXCR4 primers were designed on primer 3 and purchased from Integrated DNA Technologies (CXCR4 primer sequence, forward: ACGCCACCAACAGTCAGAG and reverse: AGTCGGGAATAGTCAGCAGGA3′). qRT–PCR analysis was carried out on three independent RNA samples.

### Proliferation assay

Stably transfected cells were seeded in triplicate in 6‐well plates (1.2 × 10^4^ cells per well). Cells were harvested and counted in triplicate over the next 5 days. Proliferation was also assessed using the 3‐(4,5‐dimethylthiazol‐2‐yl)‐2,5‐diphenyltetrazolium bromide (MTT) cell viability assay as previously described (Suh *et al*., 2015). Optical density was measured at a wavelength of 595 nm on a Tecan Infinite F50.

### Cell cycle assay

The cell lines were synchronised using two 18‐h thymidine (2 mm) blocks with an 8‐h release. 1 × 10^6^ cells were harvested and centrifuged for 5 min at 1000 ***g***. The cell pellets were fixed in 500 μL 1x PBS and 4.5 mL cold 70% ethanol overnight at 4 °C. After 24 h, the cells were centrifuged for 10 min at 1000 ***g***, and the cells were washed once with 1x PBS, treated with 300 μL of RNase A/PBS solution (1 mg·mL^−1^) and stained with propidium iodide (50 μg·mL^−1^, Sigma) solution at 37 °C for 30 min. The DNA histograms were generated with a Becton Dickinson Bioscience (BD) Aria flow cytometer.

### Scratch assay

Confluent cells were serum starved for 24 h, before a scratch was introduced using a p200 pipette tip. Floating cells were removed by washing twice with PBS before the reintroduction of media with 10% FBS. Images were taken at 0 and 8 h. The area between the scratch was imaged and analysed using ImageJ and the percentage of the scratch area closed between the time points calculated.

### Soft agar colony‐forming assay

Sterile agarose solution of 1% and 0.6% in sterile water was mixed with the same volume of 2 ×  DMEM or DMEM F‐12 with 20% FBS and used as bottom and top layers, respectively. 5x10^3^ cells in 100 μl of appropriate culture medium were added to the top layer for 21 days. Colonies were fixed with 4% paraformaldehyde and stained with 0.1 mL of Crystal violet (0.5 mg·mL^−1^ in PBS). Images were acquired with x20 objective on an Olympus BX61 microscope.

### Anoikis resistance sphere assay

Cell was disaggregated into a single‐cell suspension and plated at 500 cells·cm^−2^ into polyhema [1.2% poly(2‐hydroxyethylmethacrylate)/95% ethanol] coated 6‐well plates for 5 days. Spherical colonies >60 μm were counted over subsequent generations to discount aggregates. Normalised percentage sphere‐forming efficiency was calculated as number of spheres formed divided by the number of cells seeded and then normalised against the control.

### Matrigel transwell invasion assay

Cell was seeded at 4x10^5^/mL in serum‐free media in 12‐well transwell 300 μg·mL^−1^ Matrigel‐coated inserts for 24 h (BD Biosciences, San Jose, CA, USA). Chemoattractant (medium containing 5% FBS and medium containing 5% FBS + CXCL12) was added to the bottom chamber of each transwell. The noninvading cells in the upper chamber were removed, and the invading cells were fixed in 100% methanol, stained with 1% toluidine blue/borax solution and scored. Five representative images from each membrane were counted and invasion was expressed as the percentage invaded cell through the Matrigel membrane relative to the cells seeded.

### 3D invasion assay

The assay was performed as previously described in Berens *et al*. (2015). A cell suspension of 5 x 10^4^ was generated and hanging drop cultures made by placing 20 μL drops of cell suspension on to the lid of a 10‐cm dish using a multichannel pipette (five rows of eight drops making 40 drops). Five microlitres of sterile PBS was added to the dish to prevent the hanging drops from evaporation. The lid is inverted and incubated at 37 °C for 72 h for spheroid formation.

After 72 h, an artificial ECM mixture was created consisting of 100 μL of growth factor‐reduced Matrigel (BD Biosciences) mixed with 100 μL of type I collagen per well and maintained on ice. The spheroids were collected and transferred into a 1.5‐mL microcentrifuge tube, and the spheroids were allowed to settle for 10 min. The spheroids were aspirate from the bottom 40 μL and added to 200 μL of the ECM mixture. Forty microlitres of the spheroid/ECM mixture was added to the centre of a 24‐well plate and placed in a 37 °C incubator for 30 min to polymerise the ECM mixture. After 30 min, 1 mL of 37 °C cell culture media was added to each well slowly to prevent dislodging.

### Statistical analysis

Student's two‐tailed t‐test was performed for assessing differences between two independent groups. When measuring several independent factors between several groups, two‐way ANOVA was used. graphpad prism 5.03 was used for statistical analyses (La Jolla, CA, USA).

## Results

### miR‐9 affects cellular proliferation, cell cycle, colony formation and invasion in HNSCC

**Figure 1:**
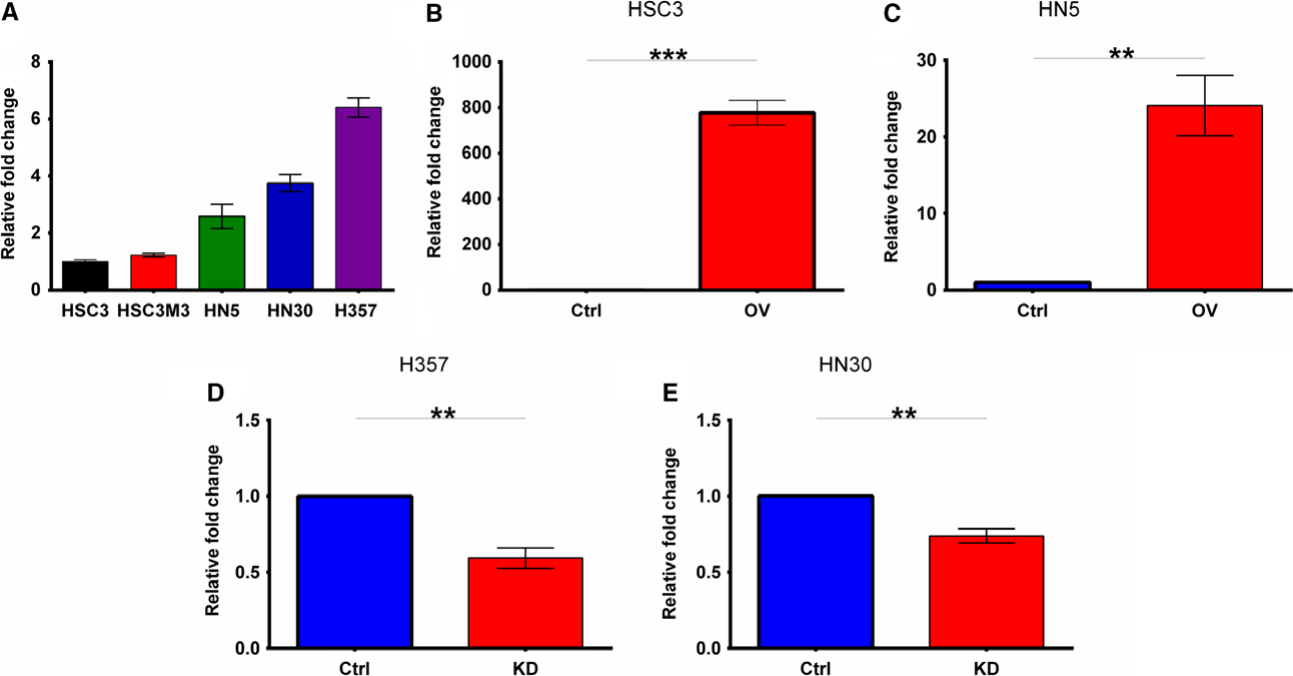
miR‐9 expression in a panel of HNSCC cell lines and the modulated cell lines. (A) Relative expression of miR‐9 in six different HNSCC cell lines using qRT–PCR normalised against HSC3. (B) HSC3 and (C) HN5 were stably transduced with miR‐9 overexpression vectors, and levels were measured by qRT–PCR (D) H357 and (E) HN30 were stably transduced with miR‐9 knockdown vectors, and the efficiency of knockdown was measured by qRT–PCR. Data represent mean ± SEM for three independent (*n *=* *3) experiments. Asterisks (*) show statistical significance as follows: *P <* *0.05, **P <* *0.01, ***P <* *0.001.

The expression of miR‐9 was investigated in five head and neck cancer cell lines (Fig. 1A), and based on miR‐9 expression levels, HSC3, HN5, HN30 and H357 were selected for further functional characterisations. In HSC3 and HN5, both of which have relatively low endogenous miR‐9 expression, miR‐9 was overexpressed, and in H357 and HN30 with relatively high endogenous miR‐9 expression levels, it was knocked down. Analysis by qRT‐PCR confirmed miR‐9 overexpression in HSC3 and HN5 by 800 (*P *<* *0.05, Fig. 1B) and 25‐fold (*P *<* *0.001, Fig. 1C), respectively, compared to vector controls. However, in H357 and HN30 cells, miR‐9 knockdown resulted in approximately 40% (*P *<* *0.001, Fig. 1D) and 22% (*P *<* *0.05, Fig. 1E) decrease, respectively, compared to scrambled controls.

**Figure 2:**
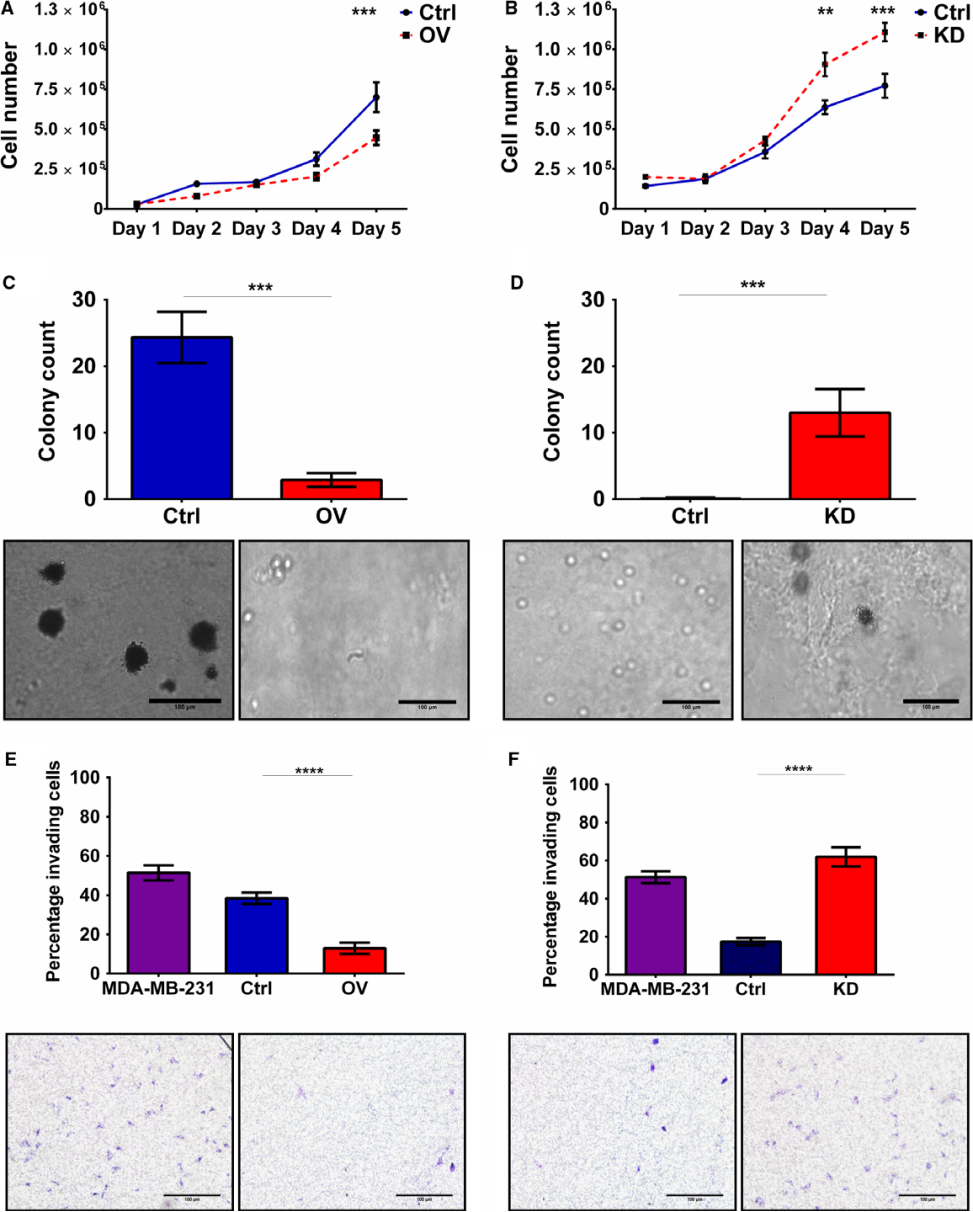
Effect of miR‐9 modulation on proliferation, cell cycle, colony formation and invasion. HSC3 and H357 cell lines were stably transfected using miR‐9 knockdown and overexpression vectors, respectively, and expression was measured by qRT–PCR. Cell proliferation of (A) miR‐9 overexpression in HSC3 and (B) miR‐9 knockdown in H357 cells was assessed by generating growth curves over 5 days. The ability of (C) HSC3 miR‐9 overexpression and (D) H357 miR‐9 knockdown cells to form colonies was tested using the soft agar assay. Invasive capacity of (E) HSC3 miR‐9 overexpression and (F) H357 miR‐9 knockdown was assessed using the Transwell Matrigel invasion assay. Representative images were taken at 10× magnification. Data represent mean number of invaded cells through the Matrigel membrane relative to migration through the control membrane. Images are representative of cells fixed and stained on the invasion membrane at 4× magnification. Scale bars = 100 μm Data represent mean ± SEM for three independent (*n *=* *3) experiments. Asterisks (*) show statistical significance as follows: *P <* *0.05, **P <* *0.01, ***P <* *0.001, ****P <* *0.0001.

Cellular proliferation experiments demonstrated suppression of cellular growth induced by miR‐9 overexpression; HSC3 miR‐9‐overexpressing cells showed a significant decrease in proliferation rate with 33% fewer cells compared to vector control on day five (*P *<* *0.01, Fig. 2A). MiR‐9 knockdown in H357 increased proliferation rate over five days (*P *<* *0.01, Fig. 2B), with a 1.42‐fold increase on day 4 and 1.44‐fold on day 5 in the miR‐9 knockdown compared to scrambled control. Immunoblotting for apoptotic markers, PARP and caspase 3, did not show differences between the miR‐9‐modulated cell lines (Fig. S1), indicating that apoptosis was not the cause of miR‐9‐mediated reduced cellular proliferation. However, cell cycle analysis demonstrated differences in the cell cycle distribution with miR‐9 knockdown H357 showing decrease in G2/M phase of the cell cycle compared to scrambled control (*P *<* *0.01, Fig. S2b) with 10.0% of the scrambled control cells in G2/M compared to 3.7% in miR‐9 knockdown cells.

Overexpression of miR‐9 in HSC3 cells resulted in a substantial ~88% reduction in colony formation in soft agar compared to vector control (*P *<* *0.001, Fig. 2C). In contrast, miR‐9 knockdown clearly resulted in the ability to form colonies in soft agar compared with no colonies formed in the scrambled control (*P *<* *0.001, Fig. 2D). Furthermore, miR‐9 overexpression resulted in a marked decrease in cellular invasion compared to control cells, the highly invasive breast cancer cell line MDA‐MB‐231 was used as a positive control (*P *<* *0.0001, Fig. 2E). Approximately 10% of miR‐9‐overexpressing HSC3 cells invaded through the membrane compared to 40% in vector controls. By contrast, invasion increased significantly by miR‐9 knockdown (*P *<* *0.0001, Fig. 2F) in H357 cells with approximately 60% of miR‐9 knockdown cells invading compared to ~20% in scrambled control cells. Similar effects of miR‐9 modulation on proliferation, cell cycle and colony formation were observed in other HNSCC cell lines (HN5 and HN30, Fig. S3).

### miR‐9 directly regulates CXCR4 expression in HNSCC

**Figure 3:**
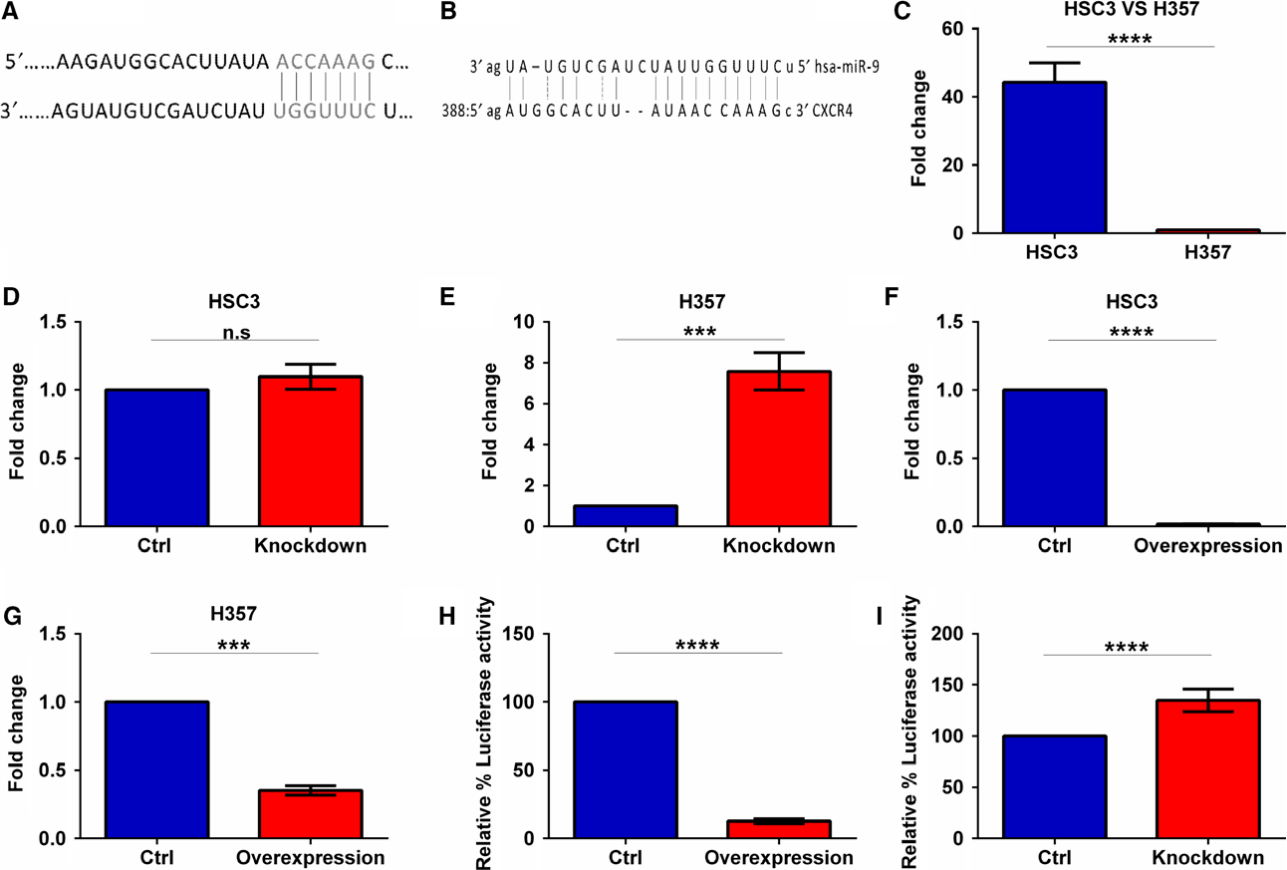
miR‐9 directly regulates CXCR4 expression. Online computer algorithm (A) TargetScan and (B) miRanda predicting CXCR4 as a miR‐9 target. qRT–PCR was performed to compare CXCR4 expression in (C) HSC3 vs H357, (D) HSC3 miR‐9 knockdown, (E) H357 miR‐9 knockdown versus the scrambled controls, whereas (F) HSC3 miR‐9 overexpression and (G) H357 miR‐9 overexpression were compared to the vector control. Luciferase assays confirming miR‐9 interaction with the 3′UTR of CXCR4 were performed for (H) miR‐9 knockdown and (I) miR‐9 overexpression. Data represent mean ±SEM for three independent (*n *=* *3) experiments. Asterisks (*) show statistical significance as follows: *P <* *0.05, **P <* *0.01, ***P <* *0.001, ****P <* *0.0001.

To identify target gene/s of miR‐9, a database (miRanda and TargetScan) search was conducted. Both algorithms indicated that miR‐9 could target evolutionarily conserved sequences in CXCR4 mRNA (Fig. 3A,B). Consequently, CXCR4 expression was analysed in HSC3 and H357 parental cell lines, scrambled controls and miR‐9 overexpression/knockdown cell lines by qRT–PCR.

Clear reverse correlation between endogenous level of miR‐9 and CXCR4 expression was detected with approximately 40‐fold increase in CXCR4 expression between the low miR‐9 expressing HSC3 and high miR‐9 expressing H357 cell lines (*P *<* *0.0001, Fig. 3C). Knockdown of miR‐9 had no significant effect on CXCR4 expression in HSC3 cells which have low endogenous miR‐9 (Fig. 3D), whereas miR‐9 knockdown in H357 resulted in over eightfold increase in CXCR4 expression (*P *<* *0.001, Fig. 3E). Notably, miR‐9 overexpression in HSC3 resulted in almost 90% decrease in CXCR4 expression (*P *<* *0.0001, Fig. 3F) and miR‐9 overexpression in H357 resulted in an approximately 60% decrease in CXCR4 level compared to controls (*P *<* *0.001, Fig. 3G).

These data show a clear link between miR‐9 and CXCR4 expression. To confirm whether miR‐9 directly regulates CXCR4 expression, a luciferase reporter construct containing the 3′‐UTR of the CXCR4 gene was introduced into miR‐9‐modulated cells. An almost 90% reduction in relative luciferase activity was detected in miR‐9‐overexpressing HSC3 cells compared to vector control (*P *<* *0.0001, Fig. 3H) and a 1.35‐fold increase in relative luciferase activity in H357 miR‐9 knockdown cells compared to scrambled control (*P *<* *0.0001, Fig. 3I). These data confirm that CXCR4 is a direct target of miR‐9 regulation in HNSCC cell lines.

### miR‐9 regulation of CXCR4 affects cellular proliferation, cell cycle, colony formation and invasion

**Figure 4:**
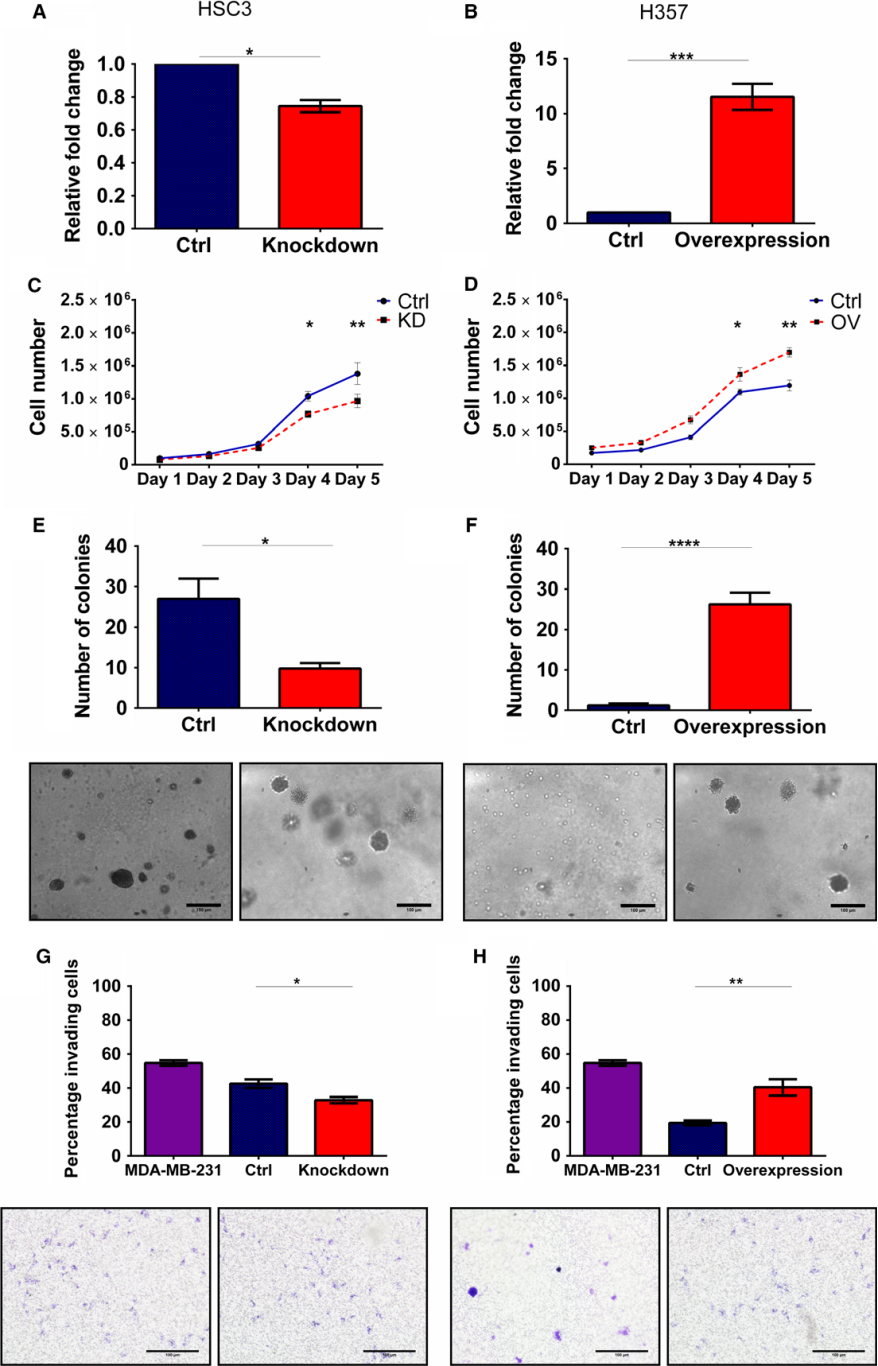
CXCR4 modulations affect cellular proliferation, cell cycle, colony formation and invasion. HSC3 and H357 were stably transfected using (A) CXCR4 knockdown and (B) overexpression vectors and analysed using qRT–PCR. Cell proliferation of (C) CXCR4 knockdown and (D) overexpression H357 cells was assessed by generating growth curves over 5 days. The ability of the (E) CXCR4 knockdown and (F) overexpression cells to form colonies was tested using the soft agar assay. Representative images were taken at 10× magnification. Invasive capacity of (G) CXCR4 knockdown and (H) overexpression cells was assessed using the Transwell Matrigel invasion assay. Representative images were taken at 10× magnification. Data represent mean number of invaded cells through the Matrigel membrane relative to migration through the control membrane. Images are representative of cells fixed and stained on the invasion membrane at 4× magnification. Scale bars = 100 μm Data represent mean ± SEM for three independent (*n *=* *3) experiments. Asterisks (*) show statistical significance as follows: *P <* *0.05, **P <* *0.01, ***P <* *0.001, ****P <* *0.0001.

Given the link between miR‐9 and CXCR4, expression of CXCR4 was modulated in HSC3 and H357 cell lines. CXCR4 knockdown in HSC3 cells resulted in almost 25% reduction in CXCR4 expression (*P *<* *0.05, Fig. 4A) whereas CXCR4 overexpression in H357 cells showed 12‐fold increase in CXCR4 expression (*P *<* *0.001, Fig. 4B). Despite partial CXCR4 knockdown in HSC3, a significant decrease in proliferation rate was observed, with about 26.1% and 29.9% decrease on days 4 and 5, respectively (*P *<* *0.01, Fig. 4C), compared to control. Whereas in H357, CXCR4 overexpression increased proliferation (*P *<* *0.01, Fig. 4D), with 1.25‐fold and 1.4‐fold more cells on days 4 and 5, respectively, in the CXCR4 overexpression cells compared to vector control. This result mirrors the effects on cellular growth observed with miR‐9 modulation.

Additionally, CXCR4 modulation had corresponding effects on cell cycle distribution: CXCR4 knockdown in HSC3 cells showed an increase in G2/M phase from 7.8% to 13.2% (*P *<* *0.01, Fig. S4a), whilst CXCR4 overexpression in H357 resulted in a statistically significant decrease in G2/M phase, from ~12% to ~5% (*P *<* *0.001, Fig. S4b).

Similar to miR‐9, CXCR4 was found to strongly affect the colony‐forming ability and invasive capacity of the HNSCC cell lines. CXCR4 HSC3 knockdown cells showed a 50% decrease in the number of colonies formed in soft agar compared to the scrambled control (*P *<* *0.05, Fig. 4E). Interestingly, CXCR4 overexpression in H357, which generally do not grow in soft agar, conferred the colony formation ability similar to the results observed with miR‐9 knockdown (*P *<* *0.0001, Fig. 4F). CXCR4 knockdown in HSC3 cells also reduced invasion capacity in these cells compared to scrambled control cells (*P *<* *0.05, Fig. 4G) whilst its overexpression induced almost twofold increase in invasion rising from 20% in controls to 40% in overexpressing cells (*P *<* *0.01, Fig. 4H). The effects of CXCR4 modulation on regulating proliferation, cell cycle and colony formation and invasion provide strong evidence for miR‐9 tumour‐suppressive effects being mediated via the inhibition of CXCR4.

### The CXCR4 ligand CXCL12 stimulates the oncogenic effects of miR‐9 knockdown

To investigate the relationship between miR‐9 and CXCR4, cellular proliferation, migration and colony formation assays were performed in the presence of CXCR4‐specific ligand CXCL12.

**Figure 5:**
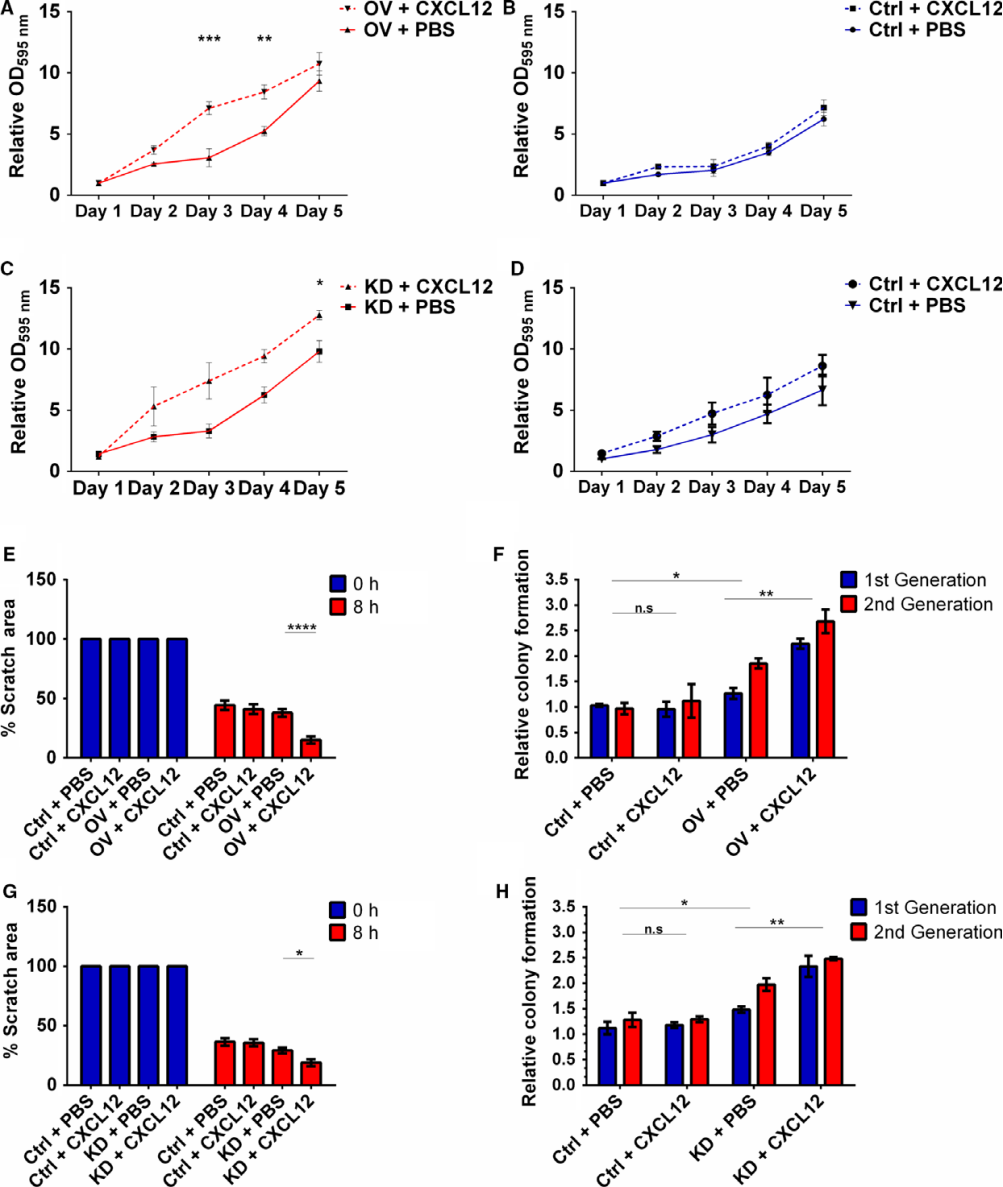
The CXCR4 ligand CXCL12 augments the oncogenic effects of miR‐9 knockdown. Cell proliferation in the presence of CXCL12 was assessed by MTT assay over 5 days in (A) CXCR4‐overexpressing and (B) vector control H357 cells. The effect of CXCL12 on cell proliferation was also assessed by MTT assay in (C) miR‐9 knockdown and (D) scrambled control H357 cells over 5 days. (E) The effect that CXCL12 stimulation of CXCR4 had on migration was examined by scratch assay using CXCR4‐overexpressing H357 cells. (F) Using sphere formation assay, the effect of CXCL12 on colony formation was investigated using CXCR4‐overexpressing H357 cells. (G) Migration of miR‐9 knockdown cells stimulated by CXCL12 was studied using scratch assay. (H) Sphere‐forming capacity of miR‐9 knockdown cells was similarly measured in the presence of CXCL12. Data represent mean ±SEM for three independent (*n *=* *3) experiments. Asterisks (*) show statistical significance as follows: *P <* *0.05, **P <* *0.01, ***P <* *0.001, ****P <* *0.0001.

As demonstrated above, CXCR4 overexpression or miR‐9 knockdown by itself increased proliferation of H357 cells. Whilst CXCL12 had no effect on cellular proliferation of the vector control cells, there was an approximately 2.33‐fold (*P *<* *0.0001) and 1.6‐fold (*P *<* *0.001) increase in proliferation on days 3 and 4, respectively, between unstimulated and stimulated CXCR4‐overexpressing cells treated with CXCL12 (Fig. 5A,B). Interestingly, miR‐9 knockdown cells had a similar response; CXCL12 had no effect on the scrambled control cells but caused an approximately 2.2‐fold (*P *<* *0.05) increase in proliferation on day 3 between stimulated and unstimulated miR‐9 knockdown cells (Fig. 5C,D).

Vector control cells showed no difference in migration after CXCL12 stimulation; however, CXCL12 stimulation induced a 2.5‐fold increase in migration in CXCR4‐overexpressing cells (*P *<* *0.05, Fig. 5E). Additionally, CXCL12 stimulation of CXCR4‐overexpressing cells resulted in an approximately 1.5‐fold increase in anoikis‐resistant spheroid colonies in low adherent conditions compared to treated and untreated controls (*P *<* *0.01, Fig. 5F).

Interestingly, wound healing assay showed that although miR‐9 knockdown by itself did not result in a change in migration of H357 cells compared to scrambled control cells, when stimulated with CXCL12, knockdown of miR‐9 induced a 1.5‐fold increase in gap closure as compared with scrambled control cells (Fig. 5G). Additionally, CXCL12 stimulation of scrambled control cells had no effect on colony formation whereas miR‐9 knockdown cells had a significant increase (over 1.2‐fold) in anoikis‐resistant spheroid colonies after CXCL12 stimulation (*P *<* *0.01, Fig. 5H). Together, these results indicate that miR‐9 knockdown similar to CXCR4 overexpression makes cells responsive to CXCL12 and strongly suggest that miR‐9 tumour‐suppressive effects are mediated via CXCR4 pathway.

### CXCR4‐specific inhibitor plerixafor mimics the tumour‐suppressive role of miR‐9

To further investigate the association between miR‐9 and CXCR4 activities in HNSCC, the CXCR4‐specific inhibitor plerixafor was used. Treatment of miR‐9 knockdown (Fig. S5a) and CXCR4‐overexpressing (Fig. S5b) cells with plerixafor showed a dose‐dependent decrease in cell viability in both groups.

**Figure 6:**
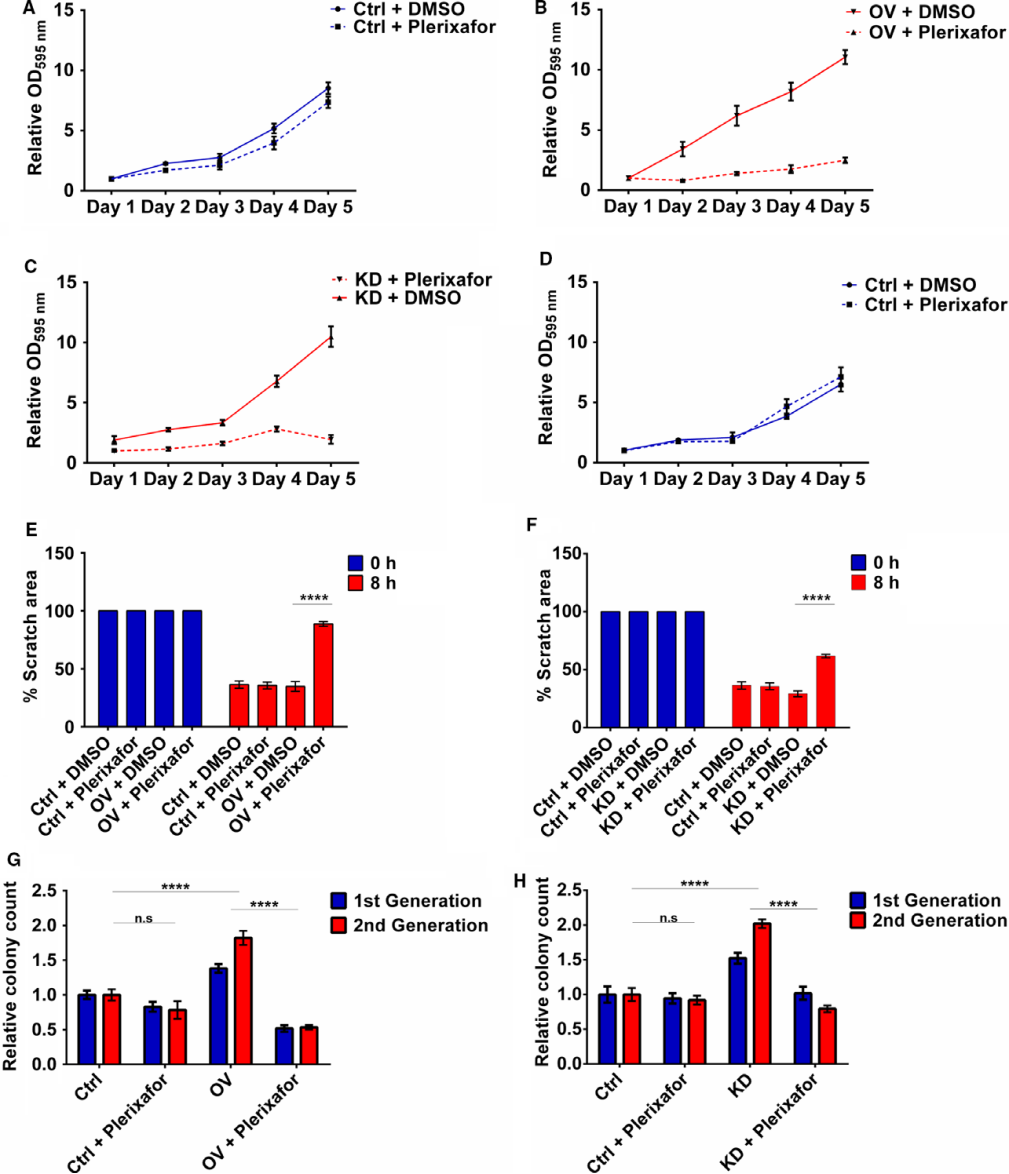
Plerixafor treatment mimics the tumour‐suppressive effect of miR‐9 via CXCR4 inhibition. Cell proliferation of (A) vector control and (B) CXCR4 overexpressing H357 cells was assessed by MTT assay over 5 days. Proliferation of (C) miR‐9 knockdown and (D) scrambled control H357 cells was also assessed by MTT assay over 5 days. (E) The effect of plerixafor on migration was examined by scratch assay using CXCR4‐overexpressing H357 cells. (F) Using sphere formation assay, the effect on colony formation was investigated using CXCR4‐overexpressing H357 cells. (G) Migration of miR‐9 knockdown cells was studied using scratch assay. (H) Sphere‐forming capacity of miR‐9 knockdown cells was similarly measured in the presence of plerixafor. Data represent mean ±SEM for three independent (*n *=* *3) experiments. Asterisks (*) show statistical significance as follows: *P <* *0.05, **P <* *0.01, ***P <* *0.001, ****P <* *0.0001.

The effect of plerixafor treatment on proliferation was investigated on CXCR4‐overexpressing and vector control cells (Fig. 6A,B). Plerixafor treatment of vector control cells had no effect on proliferation (Fig. 6A), whereas CXCR4‐overexpressing cells had a near‐complete loss in proliferation over five days (*P *<* *0.01, Fig. 6B). This effect was also observed in miR‐9 knockdown cells, with plerixafor treatment drastically reducing proliferation in only the miR‐9 knockdown cells (*P *<* *0.001, Fig. 6C,D). Interestingly, plerixafor inhibited the CXCL12 stimulated increase in proliferation in miR‐9 knockdown cells (Fig. S6).

Moreover, the inhibitory effect of plerixafor, specifically on CXCR4‐overexpressing and miR‐9 knockdown cells, was seen by changes in cell cycle profile: treatment of miR‐9 knockdown cells significantly increased G2/M progression, rising from 3.9% in untreated cells to 14.7% in treated cells (*P *<* *0.0001, Fig. S7a). Treatment of CXCR4‐overexpressing cells also resulted in a marked increase in G2/M phase compared to untreated (5.5% to 13.8%, untreated and treated; *P* < 0.0001, Fig. S7b).

Plerixafor treatment also affected migration with CXCR4‐overexpressing cells showing 50% decrease in migration with plerixafor treatment when compared to untreated and treated control cells after plerixafor treatment (*P* < 0.0001, Fig. 6E) and plerixafor treatment of miR‐9 knockdown cells resulted in 30% decrease in migration compared to untreated and treated controls (Fig. 6F).

Additionally, CXCR4 overexpression and miR‐9 knockdown in H357 increased the ability of these cells to form spheroid colonies in low adherent conditions and plerixafor treatment was sufficient to significantly block this ability. Plerixafor treatment of CXCR4‐overexpressing cells decreased spheroid formation by approximately 71.4% (p < 0.01, Fig. 6G) whilst treatment of miR‐9 knockdown cells decreased spheroid formation by about 62.5% (p < 0.0001, Fig. 6H). Collectively, these data further support miR‐9 regulating CXCR4 expression in HNSCC and suggests miR‐9 as a potential biomarker for plerixafor response.

### miR‐9 and CXCR4 regulate spheroid formation and invasion in 3D physiological conditions

Having shown acquisition of anchorage‐independent growth as well as the increased migratory and invasive capacity of HNSCC cells with miR‐9 knockdown and CXCR4‐overexpressing cells in 2D culture, we then went on to investigate these phenotypes in 3D anchorage‐independent condition.

**Figure 7:**
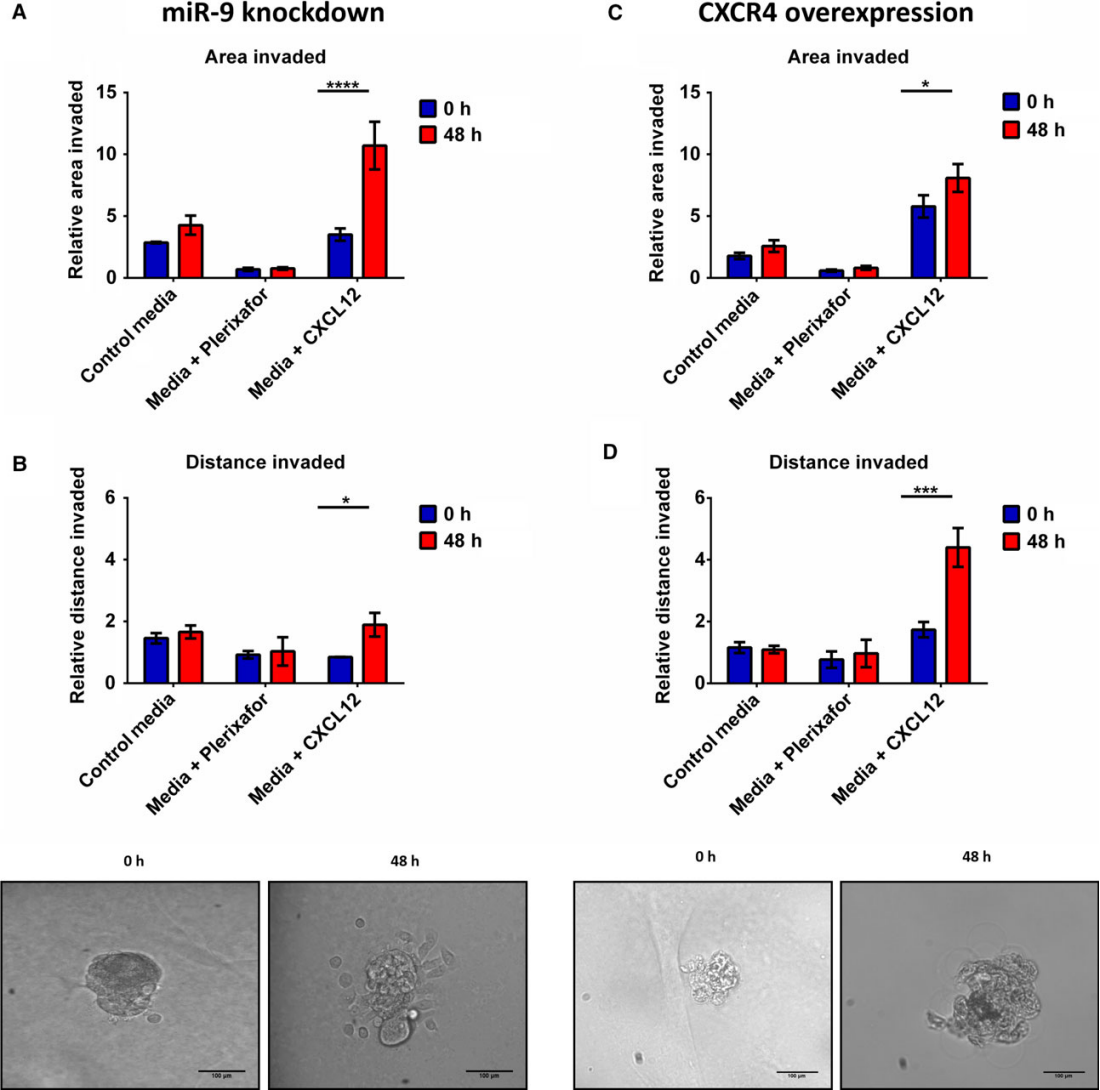
miR‐9 and CXCR4 regulate invasion under anchorage‐independent conditions. Invasion of miR‐9 knockdown cells was quantitated using imagej software. Invasion of miR‐9 knockdown cells was calculated by measuring the (A) total area invaded by the spheroid and (B) the longest invasive distance from the spheroid. Invasion of CXCR4‐overexpressing cell was also investigated by (C) total area invaded and (D) longest invasive distance. Data represent mean ±SEM for three independent (*n *=* *3) experiments. Scale bars = 100 μm Asterisks (*) show statistical significance as follows: *P <* *0.05, **P <* *0.01, ***P <* *0.001, ****P <* *0.0001.

Both miR‐9 knockdown and CXCR4‐overexpressing cells were able to form 3D spheroids in hanging drop conditions as described in the Materials and Methods section, whereas the controls cells were unable to form any spheres (data not shown). Importantly, miR‐9 knockdown and CXCR4‐overexpressing spheroids were only able to invade into the artificial extracellular matrix when treated with CXCL12 compared to normal media and media containing the inhibitor plerixafor. Spheroid invasion was assessed based on the longest invasive distance from the centre of the spheroid and the total area changed in the area invaded by the spheroid. MiR‐9 knockdown spheroids invaded more uniformly into a greater area (Fig. 7A, *P* < 0.0001) as opposed to travelling a greater distance (Fig. 7B, *P* < 0.05) over 48 h. The opposite was true for CXCR4‐overexpressing spheroids, which had a modest increase in total area invaded (Fig. 7C, p < 0.05) but travelled a significantly larger distance (Fig. 7D, *P* < 0.001) over a 48‐h period.

## Discussion

Treatment options for HNSCC have remained relatively unchanged over the past 30 years and these cancers still have high relapse and mortality rates (Mehanna and Ang, 2012). Increasing evidence indicates the importance of miRNA in different aspects of cancer development and progression (Lu *et al*., 2005). Previous data from our laboratory showed that in a group of head and neck cancer patients with good and bad clinical outcomes, miR‐9 was significantly downregulated in the patients with poor clinical outcome (Suh *et al*., 2015). Here, we used functional studies and demonstrated that knockdown of miR‐9 in HNSCC cell lines increased proliferation, cell cycle progression, colony formation and invasion. We identified CXCR4 as the important target gene of miR‐9 with CXCR4‐overexpressing cells having the reciprocal effect to miR‐9 knockdown. The direct link of miR‐9 and CXCR4 was confirmed by CXCR4 inhibitor plerixafor which reduced the oncogenic phenotype in both CXCR4‐overexpressing and miR‐9 knockdown cells. Collectively, these data indicated that miR‐9 is a tumour suppressor and implies CXCR4 as its potential oncogene target in HNSCC.

In recent years, miR‐9 has emerged as an important miRNA in cancers and its role seems to vary in different tumour types (Fenger *et al*., 2014; Hildebrandt *et al*., 2010; Lu *et al*., 2012, 2014a,b; Sun *et al*., 2013; Yu *et al*., 2014). In HNSCC, miR‐9 has been shown to be downregulated (Lujambio *et al*., 2008) with hypermethylation of miR‐9 loci being a frequent occurrence in HNSCC (Minor *et al*., 2012). The restoration of miR‐9 expression via demethylation of miR‐9 promotor sites inhibited viability of HNSCC cells (Minor *et al*., 2012). Furthermore, lower miR‐9 expression was observed in colon cancer and malignant melanoma tissues when compared to their respective normal tissue and this corresponded with increased cellular proliferation and decreased apoptosis (Bu *et al*., 2017; Cekaite *et al*., 2012). This reduction in miR‐9 is also observed in malignant melanoma cell lines and the ectopic expression of miR‐9 resulted in significant suppression of the proliferative ability of the malignant melanoma cell lines (Bu *et al*., 2017).

We found that miR‐9 knockdown promoted colony formation (Fig. 2C,D). To our knowledge, this is the first evidence of miR‐9 knockdown in HNSCC cells inducing anoikis‐resistant growth. Intratumoral injection of miR‐9 into a nasopharyngeal carcinoma cell line grafted to the mouse liver resulted in fewer microscopic and macroscopic metastases (Lu *et al*., 2014a,b). Subsequent work from the same group considered the role of miR‐9 in regulating metastasis. They identified that miR‐9 directly regulated E‐cadherin expression (Ma *et al*., 2010). E‐cadherin is a well‐established protein marker of EMT, mediating cell adhesion and loss of it promotes tumour metastasis (reviewed (Hu *et al*., 2016)). Loss of the epithelial marker E‐cadherin and gaining a mesenchymal marker such as vimentin are characteristic of a metastatic phenotype (White *et al*., 2013). Intriguingly, various studies in prostate (Dhingra *et al*., 2017), breast (Ma *et al*., 2010) and ovarian cancer (Zhou *et al*., 2017) have shown that overexpression of miR‐9 correlated with decreased E‐cadherin expression. However, in HNSCC, miR‐9 expression has been found to be downregulated (Lujambio *et al*., 2008; Minor *et al*., 2012). Therefore, it is unlikely that the tumour suppressor effects of miR‐9 in HNSCC are through E‐cadherin but potentially through another of its oncogenic targets such as CXCR4 expression (Fenger *et al*., 2014; Hildebrandt *et al*., 2010; Lu *et al*., 2012, 2014a,b; Sun *et al*., 2013; Yu *et al*., 2014).

CXCR4 expression levels are generally low or absent in most healthy tissues; however, in cancer, CXCR4 was found to be overexpressed in a variety of cancers over 23 types of cancer (Sun *et al*., 2010). Overexpression of CXCR4 has been implicated in tumour metastasis and has been found to metastasise to tissues with a high concentration of CXCL12, such as lungs, liver and bone marrow (Balkwill, 2004). Interestingly, immunohistochemistry on 79 oesophageal squamous cell carcinoma tissue samples found that the proliferation index was higher in patients samples with higher expression of CXCR4 or CXCL12 (Uchi *et al*., 2016). This effect on cell proliferation was also observed *in vitro* where inhibition of CXCR4 suppressed proliferation of synovial sarcoma cell lines (Kimura *et al*., 2016). Moreover, CXCR4 signalling has a role in cell cycle regulation. In glioblastoma, CXCL12 stimulation of CXCR4 increased cell cycle progression and EMT through expression of survivin (Liao *et al*., 2016). In mouse models, myeloid bone marrow‐derived cells gained promigratory ability when CXCR4 was stimulated by CXCL12 (Peled and Tavor, 2013). Intriguingly, we found that CXCR4 overexpression in H357 resulted in increased anoikis resistance growth (Fig. 4F). Ligand activation of CXCR4 receptor increased anoikis resistance via the upregulation of Bcl‐xL and downregulation of BMF (Kochetkova *et al*., 2009). Moreover, CXCR4 overexpression has previously been linked to increased colony formation in other cancer types (Liang *et al*., 2015). Silencing CXCR4 expression in breast cancer cell lines significantly decreased colony formation and overexpression of CXCR4 had the opposite effect (Liang *et al*., 2015). Additionally, higher CXCR4 expression increased the invasive capacity of the miR‐9 knockdown and CXCR4 overexpression cells both in 2D (Figs 2F and 4H respectively) and, interestingly, in 3D settings (Fig. 7). CXCR4 has been heavily linked to an increasingly invasive phenotype in a variety of cancers types; however, many of the studies have looked at CXCR4 invasion in only a 2D setting (Guo *et al*., 2015; Jeong *et al*., 2014; Niu *et al*., 2015; Xu *et al*., 2015; Ying *et al*., 2015). To our knowledge, this is the first experimental evidence of miR‐9 knockdown inducing an invasive phenotype via CXCR4 in 3D culture. There is one study that shows CXCR4‐induced invasion in a 3D setting, increased invasion of glioblastoma spheroids which could be abrogated through proteolytic cleavage of CXCL12 by cathepsin K (Hira *et al*., 2017).

We found that the CXCR4 inhibitor plerixafor reversed the effects of CXCR4 overexpression but most importantly also affected growth and invasiveness of miR‐9 knockdown cells in the HNSCC cell lines (Fig. 6). To our knowledge, this is the first evidence of plerixafor affecting miR‐9‐mediated cellular functions. Usage of plerixafor has shown dramatic reduction in carcinogenic phenotype induced by CXCR4 in various *in vitro* cancer studies in solid tumours such as prostate and cervical cancers (Chaudary *et al*., 2017; Conley‐LaComb *et al*., 2016), as well as lymphomas (Reinholdt *et al*., 2016). Plerixafor is already approved for the mobilisation of hematopoietic stem cells in lymphoma and multiple myeloma patients (Wagstaff, 2009). Moreover, inhibition of CXCR4 via plerixafor is in clinical trials for use with advanced pancreatic, ovarian and colorectal cancers (CAM‐PLEX NCT02179970, 2014) but not in HNSCC. Collectively, this raises the possibility of using plerixafor in combination with standard chemoradiation‐therapy for the treatment of head and neck cancers.

## Conclusion

In conclusion, the data presented here suggest that miR‐9 expression has a significant tumour suppressor role in HNSCC cells, potentially through regulation of cell cycle progression. Moreover, miR‐9 knockdown was shown to confer anoikis‐resistant colony formation capability in soft agar as well as increased invasion, and CXCR4 was identified as oncogenic target of miR‐9 in HNSCC. The ability of plerixafor to reverse the effects of the downregulation of miR‐9 on cellular proliferation, cell cycle progression, migration and colony formation indicates that miR‐9 might serve as a potential biomarker for the efficacy of plerixafor treatment.

## Author contributions

MT conceived the project idea and helped in the design of the experiments and quality assessment of the data, and with the organisation of the manuscript. HMH generated the data, HMH and NR helped in developing the theory, performing experiments and analysed and interpreted the data, HMH had large contribution in the writing of the manuscript, JG generated the necessary constructs and contributed to the data analysis. NF performed cell lines authentication and provided helpful data on all the cell lines used. All authors discussed the results and contributed to the final manuscript preparation.

## Supporting information


**Fig. S1.** miR‐9 knockdown and overexpression have no effect on apoptosis.
**Fig. S2.** miR‐9 knockdown affects cell cycle profile.
**Fig. S3.** miR‐9 modulation in HNSCC cells affects proliferation, cell cycle, colony formation and invasion.
**Fig. S4.** CXCR4 modulation in HNSCC cells affects cell cycle.
**Fig. S5.** Plerixafor titration on CXCR4 overexpressing and miR‐9 knockdown cells.
**Fig. S6.** Plerixafor blocks CXCL12 induced increase in proliferation in miR‐9 knockdown cells.
**Fig. S7.** Effect of plerixafor on cell cycle profile.
