# Supplementary material for: MicroRNA‐9 inhibits growth and invasion of head and neck cancer cells and is a predictive biomarker of response to plerixafor, an inhibitor of its target CXCR4
Source: Mol Oncol. 2018 Oct 25;12(12):2023–41. doi: 10.1002/1878-0261.12352 (PMC6275261; doi:10.1002/1878-0261.12352)
Supplement: Supplementary file 1 — Fig. S1. miR‐9 knockdown and overexpression have no effect on apoptosis. Fig. S2. miR‐9 knockdown affects cell cycle profile. Fig. S3. miR‐9 modulation in HNSCC cells affects proliferation, cell cycle, colony formation and invasion. Fig. S4. CXCR4 modulation in HNSCC cells affects cell cycle. Fig. S5. Plerixafor titration on CXCR4 overexpressing and miR‐9 knockdown cells. Fig. S6. Plerixafor blocks CXCL12 induced increase in proliferation in miR‐9 knockdown cells. Fig. S7. Effect of plerixafor on cell cycle profile. [file MOL2-12-2023-s001.pdf]

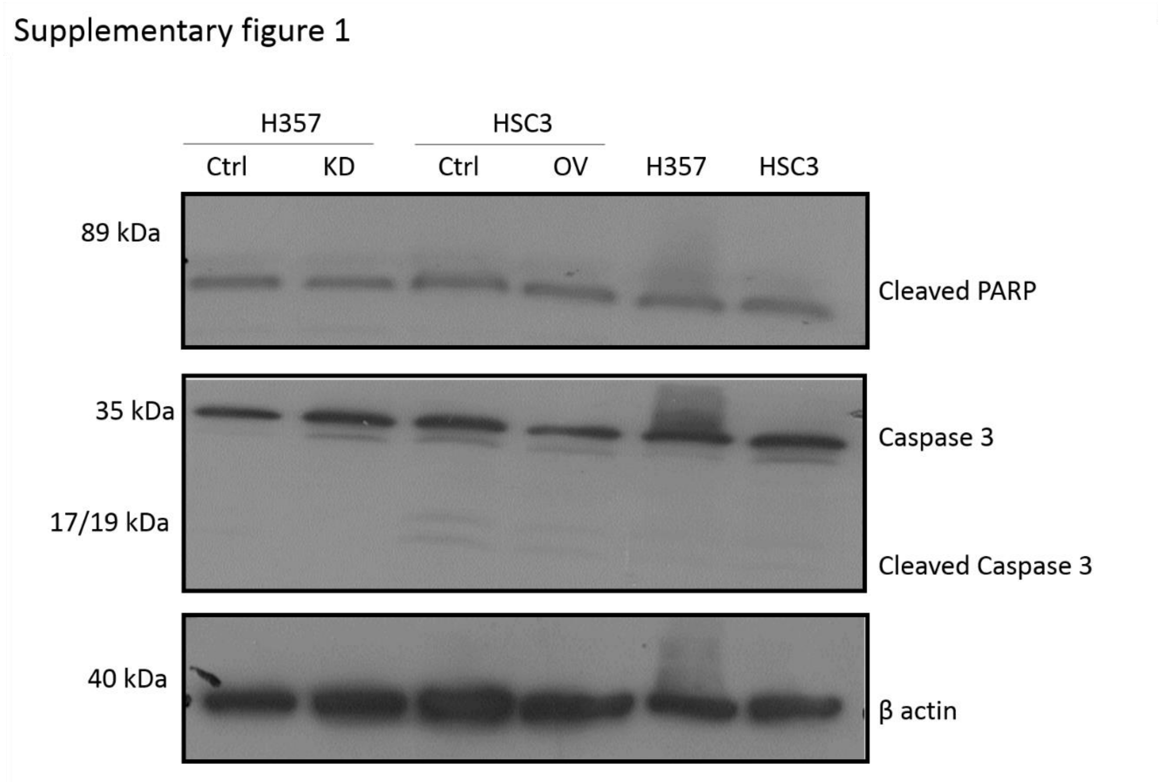

Supplementary Figure 1. **miR-9 knockdown and overexpression have no effect on apoptosis.** Expression of the apoptotic marker proteins cleaved PARP and cleaved caspase-3 was assessed by Western blotting using lysates of HSC3 and H357 parental cells and HSC3 miR-9 overexpressing and H357 miR-9 knockdown cells.

Supplementary figure 2

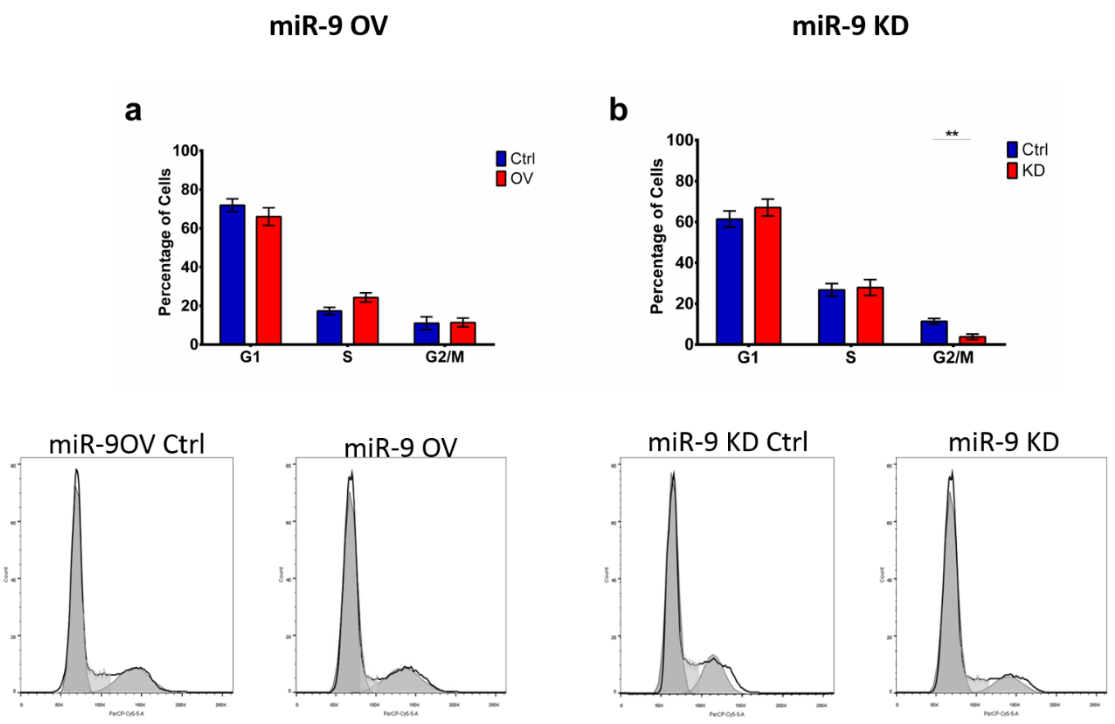

Supplementary Figure 2. **miR-9 knockdown affects cell cycle profile.** Cell cycle analysis of **a.** HSC3 miR-9 overexpression and **b.** H357 miR-9 knockdown cells was conducted using PI staining and flow cytometry. Data represents mean ±SEM for three independent (n=3) experiments. Asterisks (*) show statistical significance as follows: * = $p < 0.05$, ** = $p < 0.01$, *** = $p < 0.001$, **** = $p < 0.0001$. Representative cell cycle profiles are shown.

Supplementary figure 3

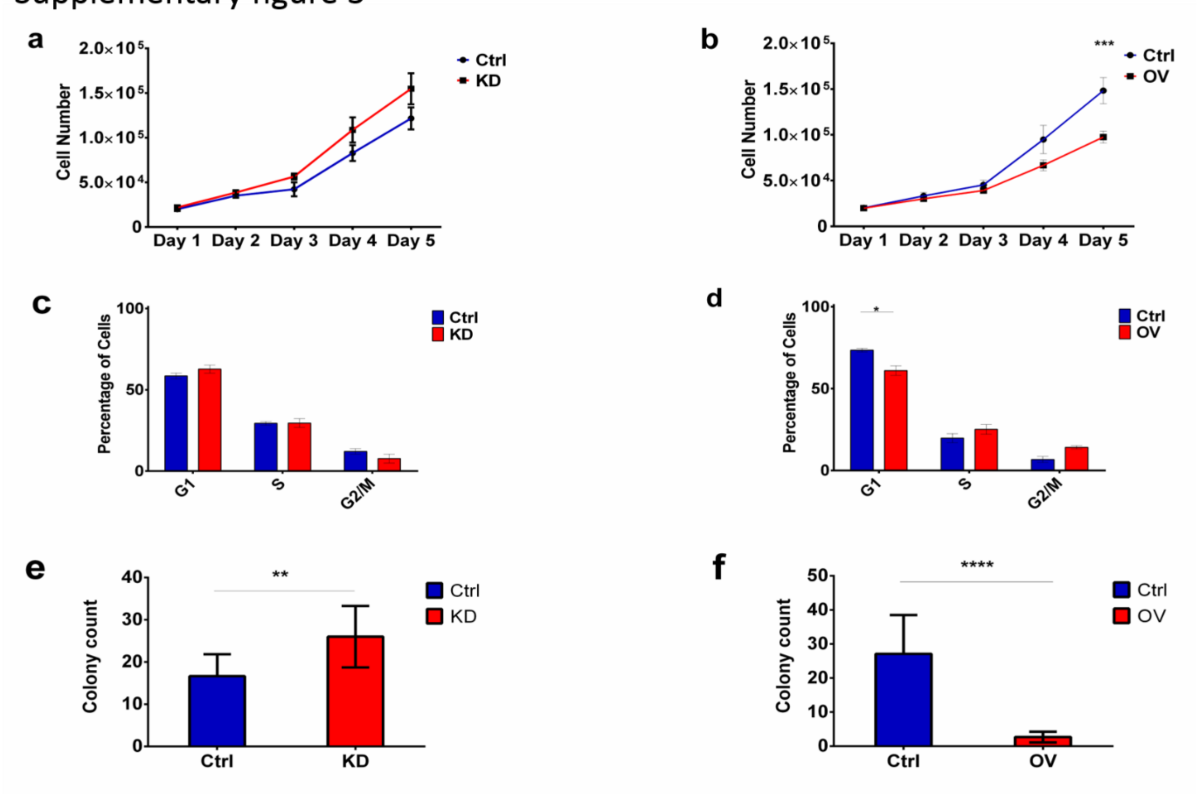

Supplementary Figure 3. **miR-9 modulation in HNSCC cells affects proliferation, cell cycle, colony formation and invasion. a. b.** Cell proliferation of HN30 and HN5 miR-9 knockdown and overexpression cells was assessed by generating growth curves over 5 days**. c. d.** Cell cycle analysis of miR-9 knockdown and overexpressing cells was conducted using PI staining followed by flow cytometry. **e. f.** The ability of the miR-9 knockdown and overexpression cells to form colonies was tested using the soft agar assay. Data represents mean ±SEM for three independent (n=3) experiments. Asterisks (*) show statistical significance as follows: * = p < 0.05, ** = p < 0.01, *** = p < 0.001, **** = p < 0.0001.

Supplementary figure 4

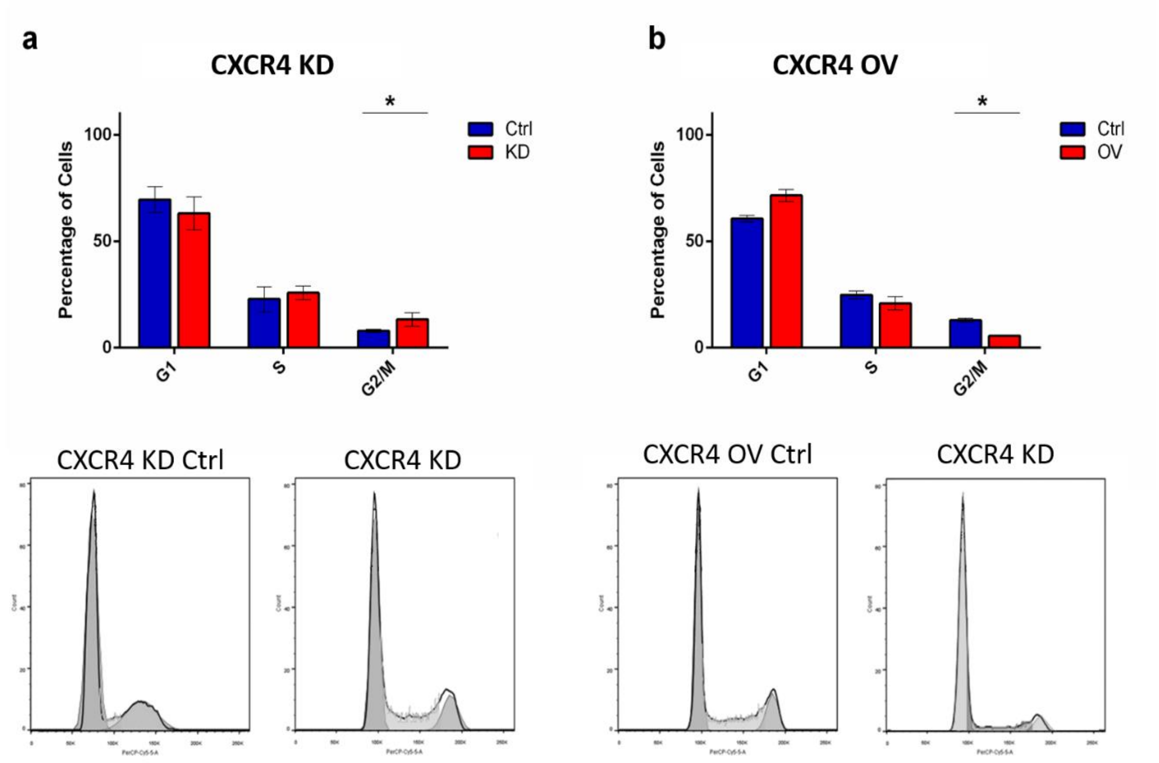

Supplementary Figure 4. **CXCR4 modulation in HNSCC cells affects cell cycle.** Cell cycle analysis of **b.** CXCR4 knockdown and **b.** overexpressing cells was conducted using PI staining and analysed by flow cytometry. Data represents mean ±SEM for three independent (n=3) experiments. Asterisks (*) show statistical significance as follows: * = p < 0.05, ** = p < 0.01, *** = p < 0.001, **** = p < 0.0001. Representative cell cycle profiles are shown.

Supplementary figure 5

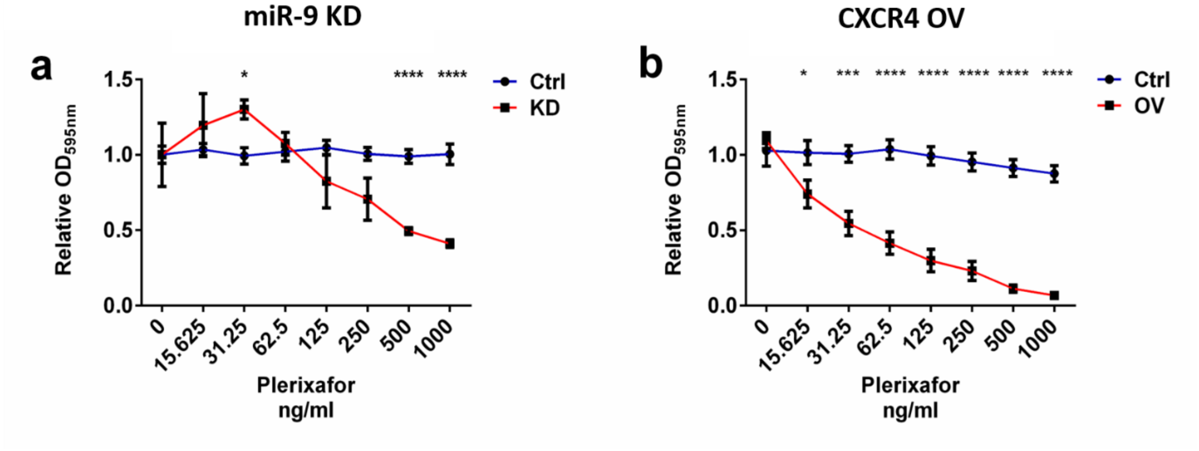

Supplementary Figure 5. **Plerixafor titration on CXCR4 overexpressing and miR-9 knockdown cells. a. b.** Effectiveness of plerixafor assessed by MTT assay in miR-9 knockdown and CXCR4 overexpression cells. Data represents mean ±SEM for three independent (n=3) experiments. Asterisks (*) show statistical significance as follows: * = $p < 0.05$, ** = $p < 0.01$, *** = $p < 0.001$, **** = $p < 0.0001$.

Supplementary figure 6

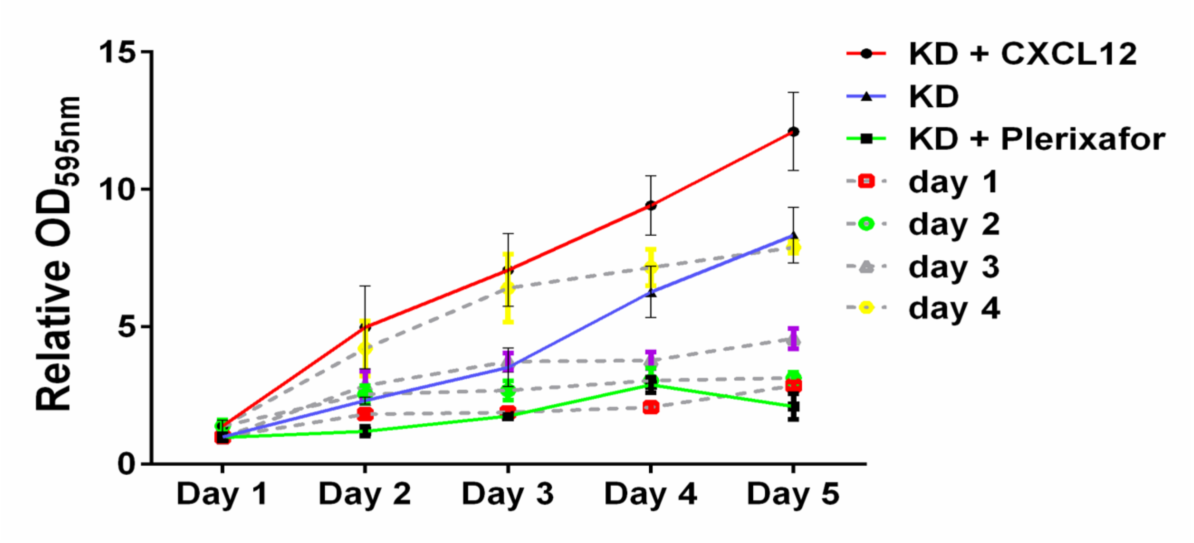

Supplementary Figure 6. **Plerixafor blocks CXCL12 induced increase in proliferation in miR-9 knockdown cells.** Cells were grown in media supplemented with CXCL12. Plerixafor was added on day 1-5 and the effect on cell growth was observed. Data represents mean ±SEM for three independent (n=3) experiments.

Supplementary figure 7

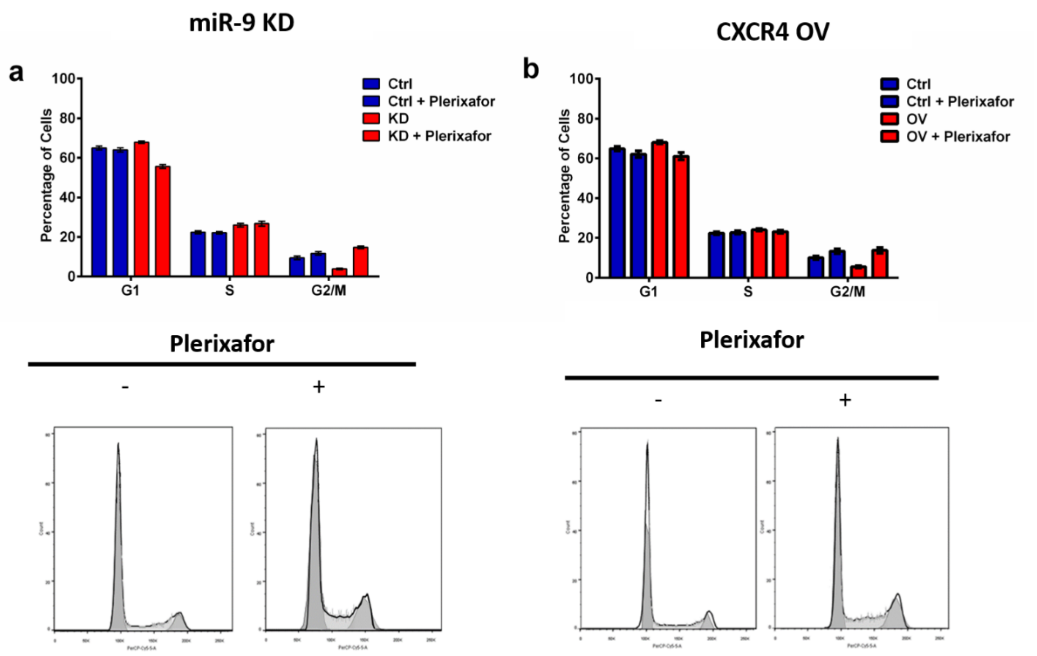

Supplementary Figure 7. **Effect of plerixafor on cell cycle profile.** Cell cycle analysis of **a.** miR-9 knockdown and **b.** CXCR4 overexpressing cells after addition of plerixafor was conducted using PI staining and analysed by flow cytometry. Data represents mean ±SEM for three independent (n=3) experiments. Asterisks (*) show statistical significance as follows: * = $p < 0.05$, ** = $p < 0.01$, *** = $p < 0.001$, **** = $p < 0.0001$. Exemplary cell cycle profiles are shown.
